# Comparative evaluation of Panther Fusion and real-time PCR for detection of *Burkholderia pseudomallei* in spiked human blood

**DOI:** 10.1099/acmi.0.000333

**Published:** 2022-03-21

**Authors:** Ian Gassiep, Michelle J. Bauer, Melissa Page, Patrick N. A. Harris, Robert Norton

**Affiliations:** ^1^​ University of Queensland Centre for Clinical Research, Royal Brisbane and Woman’s Hospital, Herston, Queensland, Australia; ^2^​ Department of Infectious Diseases, Mater Hospital Brisbane, South Brisbane, Queensland, Australia; ^3^​ Pathology Queensland, Royal Brisbane and Women’s Hospital, Herston, Queensland, Australia; ^4^​ Pathology Queensland, Townsville University Hospital, Townsville, Queensland, Australia; ^5^​ Faculty of Medicine, University of Queensland, Brisbane, Queensland, Australia

**Keywords:** melioidosis, *Burkholderia pseudomallei*, bacteraemia, molecular detection, TTS1, PCR, blood culture

## Abstract

**Introduction.** Melioidosis is an infection that most commonly presents with bacteraemia. Culture-based laboratory methods can result in a significant delay to organism identification. Molecular diagnostic techniques have a high sensitivity and rapid time to diagnosis. A decreased time to diagnosis is likely to improve patient outcomes.

**Aim.** To compare the Panther Fusion automated molecular instrument to an in-house method for the detection of *Burkholderia pseudomallei* directly from spiked human whole-blood samples.

**Results.** The in-house method detected 11/12 (92 %) samples with a *B. pseudomallei* concentration of 2.5–4.5×10^2^ c.f.u. ml^−1^. The Panther was less reliable, detecting only 8/14 (75 %) samples with a similar bacterial concentration. The Panther was able to detect 12/12 (100 %) spiked blood culture-positive samples.

**Conclusion.** The direct detection of *B. pseudomallei* from patient blood on presentation to a healthcare facility will significantly decrease time to diagnosis. We describe an in-house real-time PCR method with the lowest reported limit of detection to date. Due to lower sensitivity, the Panther Fusion would be best used as a diagnostic method directly from a positive blood culture.

## Introduction

Melioidosis is an infectious disease caused by *Burkholderia pseudomallei *, an environmental Gram-negative bacteria. The environmental niche of this organism is soil and water of tropical and subtropical regions [1]. It is currently estimated that *B. pseudomallei* is endemic in approximately 80 countries [2]. Globally, melioidosis may account for over 200 000 deaths annually [2]. Furthermore, melioidosis is estimated to account for a greater burden of disability-adjusted life years per one million population than any other World Health Organization-recognized neglected tropical disease [3].

The most common clinical presentation of melioidosis is pneumonia [1]. In a study of over 7000 patients in Thailand, 38 % were diagnosed with a pulmonary infection, and in an Australian study of over 1000 patients the rate was 52 % [4, 5]. The most common microbiological diagnosis is bacteraemia, which is present in up to 73 % of patients [6]. Melioidosis is predominantly an opportunistic infection, affecting patients with underlying risk factors, the most common of which is diabetes mellitus [1]. Culture of the organism has been considered the diagnostic gold standard, but the sensitivity of culture may be as low as 60.2 % [7]. Additionally, laboratory identification of *B. pseudomallei* can be challenging. Common clinical microbiological methods for organism identification, such as automated phenotypic analysis, have a high error rate and can misidentify the organism as a number of non-fermenting Gram-negative organisms, such as *Pseudomonas* or *Acinetobacter* species [1]. Therefore, the combination of a high rate of bacteraemia in a comorbid population coupled with a potential delay in diagnosis results in a high case fatality rate.

There have been many attempts to improve identification of *B. pseudomallei* and therefore diagnosis of melioidosis [1]. Molecular identification of *B. pseudomallei* from clinical samples was first described in 1994 [8]. Since this initial evaluation multiple genetic targets have been trialled directly from bacterial isolates and also various clinical samples [9]. The ability to accurately detect *B. pseudomallei* directly from a clinical sample greatly improves the time to diagnosis. The culture-independent nature of this technique could reduce this window period by approximately 24 h in non-blood specimens. In bacteraemic patients molecular identification directly from blood at the time of presentation could decrease time to diagnosis by more than 48 h [10, 11].

To date, the most extensively investigated molecular target for identification of *B. pseudomallei* is the type III secretion system (TTS) gene cluster 1. This is a highly conserved region in the *B. pseudomallei* genome and plays a critical role in pathogenesis [1, 12]. The TTS1 open reading frame 2 (*orf2*) target has been validated in numerous studies, with a reported analytical sensitivity of 100 % [13]. Multiple studies have assessed the sensitivity and specificity of the TTS1-*orf*2 target when performed on clinical samples, most commonly via a real-time Taqman polymerase chain reaction (qPCR) method [9, 14–16]. Two studies of spiked whole blood demonstrated 100 % sensitivity at a bacterial concentration of 5.5×10^3^ and 8.4×10^3^, respectively [14, 17]. Specificity was reported as 100 % [14]. Assessment of the buffy coat layer on clinical samples revealed a clinical sensitivity of 0–100 % [15, 16]. Notably, all patients with septic shock were PCR-positive in one study, likely in keeping with a higher bacterial burden at the time of blood collection [15].

Given the improved accuracy and potential decrease in time to diagnosis, culture-independent molecular techniques represent a means to improve patient outcomes. Previous studies have demonstrated either mixed results or detection only from high bacterial concentrations in blood [9]. Additionally, multiple automated PCR platforms are now available, some of which include the ability to use research functionality in order to perform laboratory-developed tests [18]. One such instrument is the Panther Fusion (Hologic, San Diego, CA, USA), which has recently been installed in multiple Pathology Queensland laboratories, including the melioidosis-endemic regions of Cairns and Townsville, Australia. The authors therefore aimed to evaluate the Panther Fusion (referred to below as Panther) with regard to limit of detection (LOD) in spiked human whole-blood samples as a pilot study. Furthermore, the authors aimed to compare the Panther to a semi-automated in-house PCR method that utilizes standard PCR reaction mix and cycling conditions routinely used by our laboratory.

## Methods

### Whole blood spiking

A 4 ml volume of healthy human donor blood was collected in 4 ml Vacutainer K2 EDTA tubes (BD Diagnostics, Sparks, MD, USA). A 0.5 McFarland suspension was created in sterile saline using a *B. pseudomallei* type strain, National Collection of Type Cultures (NCTC) 13 178. Whole-blood EDTA was spiked with decreasing dilutions to a final concentration of 10^3^, 10^2^ and 10^1^ colony-forming units (c.f.u.) ml^−1^. A 100 µl and 500 µl aliquot of each spiked blood sample was inoculated in duplicate onto 5 % horse blood agar and incubated at 35 °C in aerobic conditions for 48 h to assess final concentration. Additionally, two BacT/Alert FA (bioMérieux, Marcy l’Etoile, France) aerobic blood culture bottles were spiked with 9 ml of donor blood and 1 ml of ~1.1×10^3^ c.f.u. ml^−1^ *B. pseudomallei* suspension and incubated in the BacT/Alert VIRTUO automated instrument.

### Panther

The Panther is a fully automated platform and therefore DNA extraction, purification and thermal cycling occur within the instrument. As previously reported, the TTS1-*orf2* primers and probe were used [14]. Following optimization experiments (data not reported) a molecular master mix was created consisting of 5 µl 100 µM forward and reverse primers, 3.7 µl 200 µM probe, 34.5 µl potassium chloride, 4.25 µl magnesium chloride and 8.5 µl Tris buffer. Additionally, 14 and 21 µl of a proprietary internal control primer and probe were included. The final volume of 850 µl was created with purified molecular grade water, and Open Access RNA/DNA polymerase cartridges were included as per the manufacturer’s instructions. Thermocycler conditions included a 2 min 95 °C hold stage and 45 cycles comprising 8 s at 95 °C and 25 s at 60 °C.

### In-house method

Bacterial DNA from a 200 µl sample of whole blood from an EDTA tube was extracted using the MagNA Pure 96 instrument (Roche Diagnostics, Indianapolis, IN, USA). *B. pseudomallei* DNA detection from the extracted whole-blood samples was performed using the TTS1-*orf2* as previously mentioned on the Rotor-Gene Q thermal cycler using the QuantiTect Probe Master Mix (QIAGEN, Valencia, CA, USA). A 5 µl aliquot of template DNA was added to 20 µl volume of master mix. Equine herpes virus was used as an internal control for each sample. Thermocycler conditions included a 15 min 95 °C hold stage and 45 cycles comprising 15 s at 95 °C and 60 s at 60 °C.

## Results

Three separate experiments comparing the limit of detection directly from an EDTA whole-blood sample using the Panther and in-house molecular method were performed over 3 days. Both the Panther and in-house method were able to detect all samples with a concentration of 1.6–2.8×10^3^ c.f.u. ml^−1^, Table 1. The Panther was less reliable at lower concentrations, detecting only 8/14 (75 %) samples with a concentration of 2.5–4.5×10^2^ c.f.u. ml^−1^. Additionally, the Panther was unable to detect *B. pseudomallei* in samples with a concentration of 10^1^ c.f.u. ml^−1^ or lower. The in-house method demonstrated a more reproducible LOD, detecting 11/12 (92 %) samples with a *B. pseudomallei* concentration of 2.5–4.5×10^2^ c.f.u. ml^−1^. Only 6/24(25 %) samples with a concentration of 10^1^ c.f.u. ml^−1^ or lower were detected by this method.

**Table 1.** Comparison of the Panther and in-house method limit of detection

	*Run 1*								Internal control	
* B. pseudomallei* c.f.u. ml^−1^	1.7×10^3^		2.5×10^2^		2.5×10^1^		0.2×10^1^			
	PCR+	*C*_t_ mean (sd)	PCR+	*C*_t_ mean (sd)	PCR+	*C*_t_ mean (sd)	PCR+	*C*_t_ mean (sd)	PCR+	*C*_t_ mean (sd)
**Panther**	6/6	35.1 (1.8)	6/6	38.9 (2.9)	0/6	–	0/6	–	24/24	30.6 (0.4)
**In-house**	4/4	32.8 (0.3)	4/4	35.9 (1.1)	2/4	37.3 (0.7)	0/4	–	16/16	28.2 (0.3)
	***Run 2***									
*** B. pseudomallei* c.f.u. ml^−1^**	2.8×10^3^		4.5×10^2^		1.0×10^1^		0.75×10^1^			
	PCR+	*C*_t_ mean (sd)	PCR+	*C*_t_ mean (sd)	PCR+	*C*_t_ mean (sd)	PCR+	*C*_t_ mean (sd)	PCR+	*C*_t_ mean (sd)
**Panther**	4/4	39.8 (0.8)	1/4	41.7 (–)	0/4	–	0/4	–	16/16	30.9 (0.2)
**In-house**	4/4	32.4 (0.4)	4/4	35.4 (0.4)	1/4	38.7 (–)	1/4	37.1 (–)	16/16	27.6 (0.1)
	***Run 3***									
*** B. pseudomallei* c.f.u. ml^−1^**	1.6×10^3^		2.4×10^2^		1.0×10^1^		0.75×10^1^			
	PCR+	*C*_t_ mean (sd)	PCR+	*C*_t_ mean (sd)	PCR+	*C*_t_ mean (sd)	PCR+	*C*_t_ mean (sd)	PCR+	*C*_t_ mean (sd)
**Panther**	4/4	39.7 (2.1)	1/4	41.1 (–)	0/4	–	0/4	–	16/16	31.1 (0.3)
**In-house**	4/4	32.5 (0.2)	3/4	36.5 (1.9)	0/4	–	2/4	37.4 (0.4)	16/16	26.9 (0.3)

**Fig. 1.**
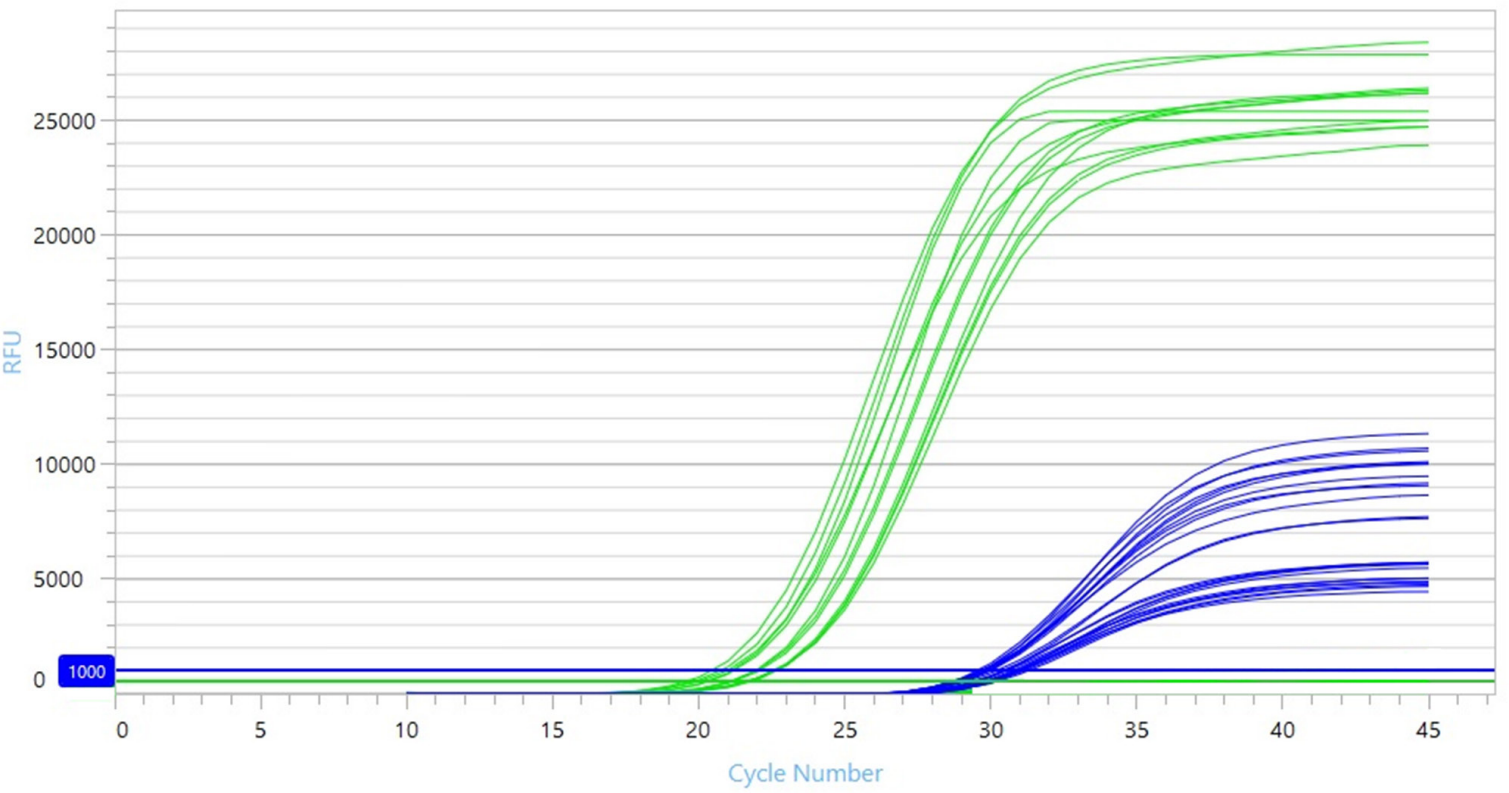
Panther amplification curves from blood culture-positive samples. Overlay of all amplification curves: *B. pseudomallei* positive blood culture samples (green) and internal controls (blue).

Finally, 12/12 (100 %) samples from two spiked positive blood culture bottles were detected using the Panther with a mean *C*_t_ value of 21.7 cycles and standard deviation (sd) 0.8, Fig. 1. Table 2 demonstrates the association between spiked organism concentration and time to detection of the blood cultures, as flagged by the Virtuo instrument.

**Table 2.** Spiked blood culture time to positive using Virtuo instrument

Inoculum	No. of spiked bottles	Spiked c.f.u. ml^−1^, mean (range)	Time to positive (hours), mean (range)
**10^3^**	3	2.8×10^3^ (1.0–4.0)	17.1 (16.5–18.0)
**10^2^**	6	3.4×10^2^ (1.0–5.6)	19.8 (16.5–22.2)
**10^1^**	5	2.7×10^1^ (1.0–6.4)	23.1 (22.2–24.9)
**10**	2	3 (1–5)	26.4 (24.7–28.0)

## Discussion

The use of molecular diagnostics in the clinical microbiology laboratory has increased over time. Currently, automated molecular diagnostic platforms are routinely used for the diagnosis of many infectious diseases, including but not limited to respiratory viruses and sexually transmitted infections [19, 20]. Automated platforms have the advantage of decreasing the time spent by laboratory scientists handling and processing samples [20]. Additionally, as each step of the sample preparation occurs within the instrument, this results in a decreased risk of sample contamination, and therefore decreased false positive or false negative results. Importantly, to a large extent, the automated platforms have removed the need for a multi-room molecular diagnostics suite within a microbiology laboratory. Finally, automated platforms have demonstrated a decrease in result turnaround time, which may have implications in areas such as antimicrobial stewardship and therefore improved patient outcomes [21].

This is the first study to assess an automated molecular diagnostic platform for the identification of *B. pseudomallei* directly from human blood. The Panther was able to identify the organism correctly in all spiked blood culture-positive samples. These results indicate that time to accurate diagnosis of melioidosis patients with bacteraemia can be greatly decreased.

The in-house method used in this analysis had a lower limit of detection and was more consistent than the Panther. This method has the ability to detect 92 % of whole-blood samples with a spiked concentration of 10^2^ c.f.u. ml^−1^. Currently, this is the lowest reported reproducibly detectable concentration using qPCR [9]. Importantly, the Panther was able to detect 100 % of samples with a concentration of ≥1.6×10^3^ c.f.u. ml^−1^. This is a better limit of detection than that of most previously reported assays [9]. The turnaround time from sample receipt to result for the Panther was approximately 3 h and for the in-house method approximately 4 h. The cost per test for the in-house method excluding labour charges is approximately US$11 compared with US$16 for the Panther (AU$ to US$ exchange rate: 0.72). While the automated method is more expensive, it is expected to significantly reduce overall labour costs due to reduced laboratory scientist hands-on time.

Panther instruments are now located in each of the microbiology laboratories in the melioidosis-endemic regions of Queensland, Australia. While previous research has demonstrated the utility of mass spectroscopy for identification of *B. pseudomallei *, these instruments are not available in all laboratories [22, 23]. Similarly, the majority of these laboratories do not have a molecular diagnostics suite and would be unable to perform the in-house method described above. Therefore, given these limitations, the use of the Panther in these facilities is more practical.

The Virtuo blood culture time to positive data aid in understanding the possible burden of infection at the time of patient presentation. A previous analysis of bacteraemic melioidosis patients reported a median bacterial concentration of 1.1 c.f.u. ml^−1^ (interquartile range, 0.2–7.7 c.f.u. ml^−1^) at time of presentation [24]. Additionally, a review of automated instrument time to positive blood culture detection for bacteraemic patients reported 62.5 % positive within 24 h [10]. Compared to the older automated instrument used in that study, the Virtuo may have a shorter time to detection of up to 2 h [25]. Our data reveal an estimated time to detection of 23 h for patients presenting with a mean bacterial concentration of 2.7×10^1^ c.f.u. ml^−1^. This suggests that the median bacterial concentration of bacteraemic patients from the previously mentioned positive blood culture study may be between 10^1^–10^2^ c.f.u. ml^−1^. The bacterial concentration in the blood of bacteraemic patients at the time of presentation has consequences for the likelihood of early detection. This study indicates that an assay with a LOD of >1.0×10^1^ c.f.u. ml^−1^ is likely to detect the majority or bacteraemic patients on presentation. While creating such an assay is a difficult task, it is 10-fold higher than previously thought [24].

There are a number of limitations with this study. Firstly, only three experiments were performed over 3 days due to a limitation of reagents. The authors note that determining a reportable range requires five–seven concentrations with two replicates each [26]. However, a protocol using high, medium and low concentration samples in duplicate has been proposed [26]. Only one *B. pseudomallei* strain was used in the experiments. As the TTS1-*orf2* target has been assessed extensively on over 1000 isolates, an additional evaluation of specificity is not warranted. However, it is possible that additional similarly specific *B. pseudomallei* molecular targets may have an improved sensitivity. Finally, as a pilot study, the evaluation was only performed on spiked human blood samples. A robust validation study using multiple clinical samples as mentioned above is required.

## Conclusion

The direct detection of *B. pseudomallei* from patient blood on presentation to a healthcare facility will significantly decrease time to diagnosis. To date, multiple molecular methods with variable performance have been published. We describe an in-house qPCR method with the lowest LOD reported. This is the first study to demonstrate the utility of an automated molecular diagnostic instrument for the diagnosis of melioidosis directly from spiked human blood samples. Currently, the Panther would be best positioned as a diagnostic method directly from a positive blood culture.
